# Case Report: Short-term disease control with subsequent icotinib therapy in EGFR L858R/C797S, T790M-negative lung adenocarcinoma after osimertinib resistance

**DOI:** 10.3389/fonc.2026.1911991

**Published:** 2026-07-20

**Authors:** Shihang Song, Di Yao, Jinlong Liu, Wenjing Nie, Mengjia He, Jianqiao Zhang, Jing Zhao

**Affiliations:** 1 Graduate School, Hebei North University, Zhangjiakou, China; 2 Department of Oncology, Hebei General Hospital, Shijiazhuang, China; 3 Graduate School, Hebei Medical University, Shijiazhuang, China

**Keywords:** C797S, EGFR mutation, icotinib, non-small cell lung cancer, osimertinib resistance, subsequent therapy, T790M-negative

## Abstract

Osimertinib resistance, particularly EGFR C797S without T790M, lacks established treatment strategies. We report a 71-year-old woman with stage IVB lung adenocarcinoma harboring EGFR L858R (T790M-negative) who received first-line osimertinib for 29 months until progression. Post-progression ctDNA revealed L858R/C797S (T790M-negative) who received first-line osimertinib for 29 months until progression. Post-progression ctDNA revealed L858R/C797S (T790M-negative). She was subsequently treated with icotinib 125 mg tid, achieving stable disease for 6 months. This case suggests that first-generation EGFR-TKI subsequent therapy may provide short-term disease control in this rare molecular context.

## 1 Introduction

Lung cancer is the most common malignant tumor worldwide in terms of both incidence and mortality, with non-small cell lung cancer (NSCLC) accounting for approximately 85% of cases (1). Epidermal growth factor receptor (EGFR) mutations are one of the most important driver mutations in NSCLC, with a prevalence of up to 40%–50% in Asian populations (2).The introduction of EGFR tyrosine kinase inhibitors (TKIs) has significantly improved the prognosis of patients with advanced EGFR-mutation-positive NSCLC (3).In patients with advanced NSCLC harboring EGFR exon 19 deletions or exon 21 L858R mutations, EGFR-TKIs have substantially improved objective response rates and survival outcomes. Osimertinib, a third-generation EGFR-TKI, irreversibly inhibits both sensitizing EGFR mutations and the T790M resistance mutation and has favorable central nervous system activity (4). It has therefore become an important first-line targeted therapy for advanced EGFR-mutant NSCLC.

Despite the established efficacy of osimertinib, acquired resistance remains unavoidable. Previous studies have demonstrated that the mechanisms of osimertinib resistance are highly heterogeneous and can be broadly classified as EGFR-dependent and EGFR-independent. EGFR-dependent mechanisms include secondary EGFR mutations such as C797S, L718Q, and G724S, whereas EGFR-independent mechanisms include MET amplification, HER2 alterations, PI3K/AKT pathway abnormalities, gene fusions, and histological transformation (5). Among these, C797S disrupts the covalent interaction between osimertinib and the cysteine residue at position 797 of EGFR and represents one of the most characteristic on-target mechanisms of resistance to third-generation EGFR-TKIs (6).

Importantly, the therapeutic implications of C797S depend on the T790M status and allelic configuration. When C797S coexists with T790M, whether the two mutations occur in cis or in trans directly affects subsequent EGFR-TKI combination strategies (7). In contrast, in a T790M-negative setting, first-generation reversible EGFR-TKIs may retain partial inhibitory activity, although the available clinical evidence remains limited and is largely derived from case reports and small observational series (8). Here, we report a patient with advanced lung adenocarcinoma harboring EGFR L858R who developed EGFR L858R/C797S, T790M-negative resistance after first-line osimertinib and subsequently achieved approximately 6 months of disease control with subsequent icotinib therapy. This case provides a clinical observation that may inform subsequent treatment for this rare molecular subtype.

## 2 Case presentation

A 71-year-old woman who had never smoked and had no family history of lung cancer presented in March 2023 with a 2-week history of chest pain and chest tightness. Physical examination showed a generally preserved condition, decreased breath sounds in the left lung, and an Eastern Cooperative Oncology Group (ECOG) performance status score of 1. Contrast-enhanced chest computed tomography (CT) demonstrated a soft-tissue nodule in the apicoposterior segment of the left upper lobe, measuring approximately 3.0 cm in maximum diameter, accompanied by multiple enlarged left supraclavicular and mediastinal lymph nodes, osteolytic destruction of the right fourth posterior rib, and thickening of the right pulmonary interlobular septa suggestive of lymphangitic carcinomatosis.

The patient underwent CT-guided lung biopsy, and pathological examination confirmed lung adenocarcinoma. Based on the radiological findings, the clinical stage was IVB (cT1cN3M1c, according to the 8th edition of the American Joint Committee on Cancer staging system). NGS of tumor tissue revealed an EGFR exon 21 L858R missense mutation (VAF, 1.54%), without EGFR T790M or other clearly defined resistance-associated mutations. (The tumor cell percentage in the biopsy specimen was approximately 30% by pathological assessment, with adequate DNA quality for NGS analysis. The relatively low VAF likely reflects intratumoral heterogeneity or dilution by stromal components, a common phenomenon in CT-guided biopsy specimens. The presence of EGFR L858R was subsequently confirmed by ctDNA testing at progression (VAF 4.16%), supporting the reliability of the initial finding).

In April 2023, the patient started oral osimertinib at a dose of 80 mg once daily as monotherapy. The treatment was well tolerated. In October 2024, follow-up contrast-enhanced chest CT showed that the primary lesion in the left upper lobe had decreased to 1.9 cm × 1.2 cm, and the response was assessed as partial response (PR) according to Response Evaluation Criteria in Solid Tumors (RECIST) version 1.1.

In September 2025, the patient developed worsening left-sided chest pain. Repeat contrast-enhanced chest CT showed enlargement of the left upper lobe lesion to 3.5 cm × 2.4 cm, and progressive disease (PD) was clinically confirmed. The PFS from the initiation of osimertinib to this progression event was approximately 29 months.

To clarify the mechanism of osimertinib resistance, peripheral blood was collected for ctDNA-based NGS (AcornMed, Xiangyi Plus-808 Gene Panel). The results showed EGFR exon 21 L858R (VAF, 4.16%), EGFR exon 20 C797S (NM_005228 exon20: c.2390G>C, p.C797S, VAF, 1.02%), and a TP53 S241F (VAF, 1.45%), with no EGFR T790M detected (defined as “not detected” with a limit of detection of 0.5% VAF). Bypass resistance mechanisms including MET amplification, HER2 amplification, ALK/RET/ROS1 fusions, BRAF V600E, and PIK3CA mutations were all negative. Because post-progression tissue rebiopsy was not obtained, as the patient declined the procedure due to concerns regarding procedural risks, it was not possible to further determine the intratumoral clonal proportion of C797S, the allelic configuration between C797S and other EGFR mutations, or the presence of histological transformation or bypass resistance mechanisms.

Considering the molecular resistance profile, the patient’s performance status (ECOG 1, age 71 years), and the limited evidence in the literature supporting first-generation EGFR-TKI subsequent therapy in the context of C797S/T790M-negative resistance, the treatment regimen was changed in September 2025 to oral icotinib hydrochloride at 125 mg three times daily. Platinum-based chemotherapy was discussed but declined by the patient due to concerns about adverse effects. Icotinib was selected because it is reimbursed by China’s National Health Insurance and was listed as a “sensitive/1A-level” recommended drug in the genetic testing report. After treatment, the patient’s chest pain improved. In November 2025, follow-up chest CT showed that the primary lesion in the left upper lobe measured approximately 3.1 cm × 2.3 cm; bone metastases remained stable, and no definite new lesions were observed. The best response was evaluated as stable disease (SD) according to RECIST version 1.1.

In March 2026, repeat chest CT showed further enlargement of the left upper lobe lesion to approximately 3.2 cm × 2.9 cm. Based on lesion morphology, symptoms, and the overall radiographic findings, renewed disease progression was determined by comprehensive clinical assessment. The PFS from the initiation of icotinib to this second progression event was approximately 6 months (**Figures 1**, **2**).

**Figure 1:**
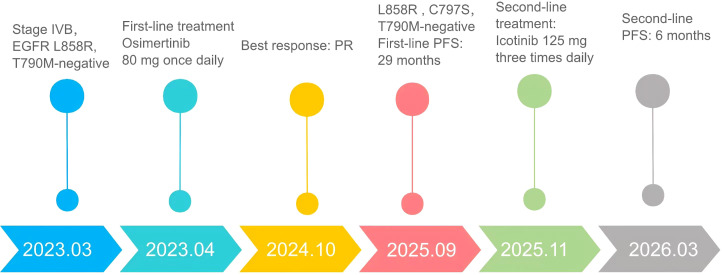
Clinical timeline of the patient. Key clinical events, treatments, and responses from diagnosis (March 2023) to progression after icotinib (March 2026). The patient achieved partial response (PR) on first-line osimertinib (80 mg once daily) with a progression-free survival (PFS) of 29 months. After progression, ctDNA revealed compound EGFR L858R/C797S mutations with T790M-negative status. Second-line icotinib (125 mg three times daily) subsequent therapy provided stable disease (SD) for 6 months. Abbreviations: PR, partial response; SD, stable disease; PFS, progression-free survival; ctDNA, circulating tumor DNA.

**Figure 2:**
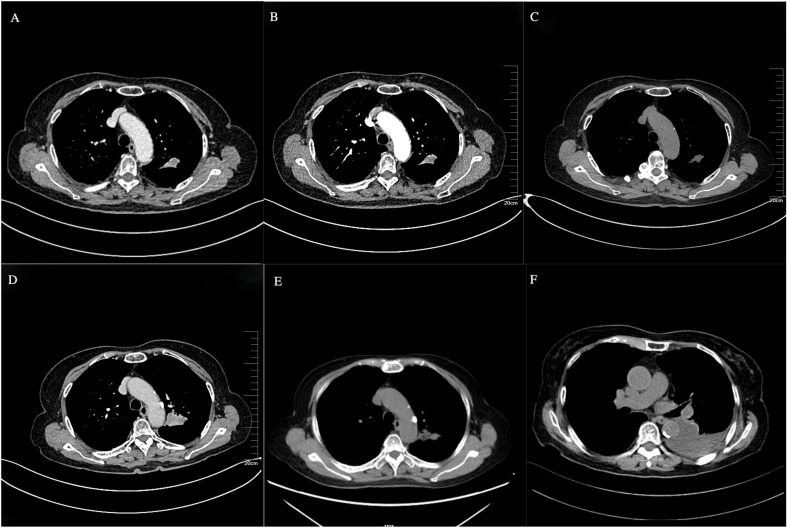
Serial chest computed tomography (CT) images during treatment. **(A)** Initial CT at diagnosis (March 2023) showing a 3.0 cm nodule in the left upper lobe. **(B)** After 18 months of osimertinib (October 2024), the lesion decreased to 1.9 × 1.2 cm, consistent with partial response (PR). **(C)** At progression (September 2025), the lesion enlarged to 3.5 × 2.4 cm. **(D)** After 2 months of icotinib subsequent therapy (November 2025), the lesion was stable at 3.1 × 2.3 cm. **(E)** At second progression (March 2026), the lesion further enlarged to 3.2 × 2.9 cm. **(F)**.

## 3 Discussion

The main clinical value of this case lies in the following observations: the patient had advanced lung adenocarcinoma harboring EGFR L858R and achieved nearly 30 months of disease control with first-line osimertinib; after progression, ctDNA revealed compound EGFR L858R/C797S mutations with a T790M-negative status; and under this molecular background, subsequent therapy with the first-generation EGFR-TKI icotinib resulted in approximately 6 months of stable disease. This treatment course provides clinically relevant evidence for individualized management after osimertinib resistance in patients with C797S/T790M-negative disease, particularly those with the L858R/C797S genotype.

Osimertinib exerts irreversible inhibitory activity by forming a covalent bond with the cysteine residue at position 797 within the EGFR kinase domain (9). When the cysteine residue at this site is replaced by serine, the covalent interaction between osimertinib and EGFR is impaired, leading to on-target resistance (10). In contrast to third-generation irreversible EGFR-TKIs, first-generation EGFR-TKIs are primarily reversible ATP-competitive inhibitors, and their activity does not depend on covalent binding to C797. Therefore, in the absence of concomitant T790M, tumor cells harboring C797S may retain partial sensitivity to first-generation EGFR-TKIs.

For patients with T790M loss or T790M-negative, C797S-positive disease, a small number of reports have suggested that reversible EGFR-TKIs such as gefitinib and erlotinib may provide transient tumor response or disease control (**Table 1**). The absence of T790M after progression in the present case may represent an important molecular basis for the observed benefit from icotinib subsequent therapy. As a gatekeeper mutation, T790M increases the affinity of EGFR for ATP and reduces the inhibitory activity of first-generation EGFR-TKIs (10). Conversely, in a T790M-negative setting, the relative disadvantage of first-generation TKIs in competing with ATP may be attenuated (16). In this case, the VAF of C797S was 1.02%, which was lower than that of concurrent EGFR L858R. This finding suggests that C797S may have represented a low-frequency resistant subclone. However, ctDNA VAF can be influenced by tumor burden, DNA shedding capacity of individual lesions, copy-number alterations, and assay platform; therefore, it cannot be directly equated with the true clonal proportion within tumor tissue (17). Accordingly, the relationship between low-frequency C797S and clinical benefit should be regarded as a plausible hypothesis rather than a definitive causal conclusion.

**Table 1:** Summary of case reports on first-generation EGFR-TKI subsequent therapy after third-generation EGFR-TKI resistance.

Case source/ year	Age/ sex	EGFR sensitive mutation	Subsequent therapy third-generation TKI	Key post-resistance mutations (T790M status)	Subsequent strategy	Best response	Rechallenge PFS
Present case/ 2026	71 / F	L858R	Osimertinib	L858R/C797S, T790M-negative	Icotinib 125 mg tid	SD	6 months
Jin et al./2025 (11)	55/F	Exon 19 deletion	Aumolertinib	Exon 19 deletion/C797S, T790M-negative	Icotinib 250 mg tid	PR → SD	8 months
Cai et al./2024 (12)	68/F	Exon 19 deletion	Osimertinib	Exon 19 deletion/C797S	Icotinib	NS	8 months
Enrico et al./2023 (13)	66/M	Exon 19 deletion + de novo T790M	Osimertinib	Exon 19 deletion/C797S, T790M-loss	Gefitinib 250 mg qd	PR	4 months
Rangachari et al./2019 (14)	52/F	Exon 19 deletion	Osimertinib	EGFR exon 19 deletion/C797S, T790M-negative	Erlotinib + Gefitinib	SD	4 months
Tan et al./2025 (15)	72/M	Exon 19 deletion	Osimertinib	EGFR 19del/C797S, T790M-negative	Gefitinib + bevacizumab	PR	15.5 months

This table summarizes published case reports of patients who received first-generation EGFR-TKIs (icotinib, gefitinib, or erlotinib) after progression on third-generation EGFR-TKIs (osimertinib or aumolertinib), with documented EGFR C797S mutations and known T790M status. Key data extracted include EGFR sensitive mutation, resistant third-generation TKI, post-resistance mutation profile (T790M status), subsequent treatment strategy, best response, and subsequent therapy progression-free survival (PFS). The present case is included for comparison. PFS, progression-free survival; PR, partial response; SD, stable disease; NS, not specified; tid, three times daily; qd, once daily; TKI, tyrosine kinase inhibitor; del, deletion.

Previous studies and case reports have shown that the therapeutic significance of C797S-mediated osimertinib resistance differs according to the T790M status. If T790M and C797S are in trans, combined first- and third-generation EGFR-TKI therapy may have theoretical activity; if they are in cis, currently available first- and third-generation EGFR-TKIs are generally ineffective (18). For patients with T790M loss or T790M-negative, C797S-positive disease, a small number of reports have suggested that reversible EGFR-TKIs such as gefitinib and erlotinib may provide transient tumor response or disease control (13). In the present case, icotinib achieved a PFS of approximately 6 months, which is broadly consistent with the theoretical rationale and clinical observations that first-generation EGFR-TKI subsequent therapy may confer short-term benefit in the C797S/T790M-negative setting.

Previous reports of first-generation EGFR-TKI subsequent therapy after emergence of C797S have mostly involved tumors with EGFR exon 19 deletion, whereas clinical data on the L858R/C797S background remain relatively limited. EGFR exon 19 deletion and L858R differ in EGFR-TKI sensitivity, co-mutation patterns, and evolutionary trajectories of resistance (19). Therefore, the present case adds clinical evidence for the rare molecular subtype of L858R/C797S with a T790M-negative status.

A TP53 co-mutation was also detected in this case. TP53 co-mutations are relatively common in EGFR-mutant NSCLC and have been associated in previous studies with shorter PFS and overall survival, potentially reflecting greater genomic instability and more aggressive tumor biology (20). However, the prognostic significance of TP53 mutations may be influenced by mutation site, functional consequence, clonal proportion, and concurrent genomic events (21). In this patient, despite the presence of a TP53 co-mutation, first-line osimertinib achieved a PFS of nearly 30 months, and icotinib provided approximately 6 months of disease control. This suggests that TP53 co-mutation should not be used as the sole factor to preclude individualized targeted therapy attempts, although it should be considered when estimating treatment expectations and planning follow-up intensity.

Renewed progression after icotinib treatment may have resulted from continuous clonal selection pressure (22). First-generation EGFR-TKIs may suppress a subset of EGFR-dependent clones but are unlikely to provide durable control of all resistant subclones (23). With ongoing treatment, further expansion of C797S-positive clones, re-emergence of T790M, acquisition of other secondary EGFR mutations, MET amplification, activation of the PI3K/AKT pathway, or histological transformation may all contribute to subsequent progression (24). Unfortunately, no tissue biopsy or repeat ctDNA results have been obtained since the disease progressed in this case, so the specific mechanism underlying the resistance to icotinib remains unclear.

This case has several limitations. First, only peripheral blood ctDNA testing was performed after progression, and post-progression tissue rebiopsy was not obtained (the patient declined the procedure). Therefore, the intratumoral distribution of C797S, cis/trans allelic configuration, histological transformation, and bypass resistance mechanisms could not be clarified. Second, ctDNA VAF does not fully represent the true clonal proportion within tumor tissue; thus, the association between low-frequency C797S and benefit from icotinib can only be proposed as a hypothesis. Third, this is a single-case observation without functional experimental validation and cannot be regarded as a standard treatment recommendation. Additionally, it remains unclear whether this approach would be effective in patients with other EGFR mutation subtypes or different T790M configurations.

This case offers several implications for clinical practice. First, after osimertinib resistance, tissue rebiopsy and/or dynamic ctDNA monitoring should be performed whenever feasible to distinguish EGFR-dependent from EGFR-independent resistance mechanisms. Second, for patients with C797S-positive disease, particular attention should be paid to the T790M status and C797S allelic configuration. Third, when T790M is absent and there is no clear evidence of bypass-driven resistance, subsequent therapy with a first-generation EGFR-TKI may be considered as an individualized treatment option, provided that the limited level of evidence is fully discussed and treatment response is closely monitored. Fourth, for patients with oligoprogressive disease, local therapy combined with continued targeted therapy may be considered; for systemic progression or progression driven by bypass alterations, platinum-based chemotherapy, antiangiogenic therapy, combined targeted therapy against bypass alterations, or enrollment in clinical trials should be considered.

This case has several limitations. First, only peripheral blood ctDNA testing was performed after progression, and post-progression tissue rebiopsy was not obtained. Therefore, the intratumoral distribution of C797S, cis/trans allelic configuration, histological transformation, and bypass resistance mechanisms could not be clarified. Second, ctDNA VAF does not fully reprt the true clonesenal proportion within tumor tissue; thus, the association between low-frequency C797S and benefit from icotinib can only be proposed as a hypothesis. Third, this is a single-case observation without functional experimental validation and cannot be regarded as a standard treatment recommendation.

## 4 Conclusion

This case demonstrates that in a patient with advanced lung adenocarcinoma harboring EGFR L858R, emergence of EGFR C797S with a T790M-negative status after first-line osimertinib resistance may be followed by short-term disease control with subsequent therapy using the first-generation EGFR-TKI icotinib. The potential biological basis may include the lack of dependence of first-generation TKIs on covalent binding to the C797 residue, partial restoration of reversible ATP-competitive inhibition in the absence of T790M, and a transient window of control created by tumor clonal heterogeneity. This case provides a clinical clue for the treatment of osimertinib-resistant L858R/C797S, T790M-negative disease; however, because the evidence is derived from a single case and lacks validation by tissue rebiopsy, further confirmation in real-world cohorts and mechanistic studies is warranted (**Table 1**).

## Data Availability

The original contributions presented in the study are included in the article/supplementary material, further inquiries can be directed to the corresponding author.
